# Functional network alterations in young brain tumor patients with radiotherapy-induced memory impairments and vascular injury

**DOI:** 10.3389/fneur.2022.921984

**Published:** 2022-09-12

**Authors:** Melanie A. Morrison, Sadie Walter, Sabine Mueller, Erin Felton, Angela Jakary, Schuyler Stoller, Annette M. Molinaro, Steve E. Braunstein, Christopher P. Hess, Janine M. Lupo

**Affiliations:** ^1^Department of Radiology and Biomedical Imaging, University of California, San Francisco, San Francisco, CA, United States; ^2^College of Osteopathic Medicine, Pacific Northwest University of Health Sciences, Yakima, WA, United States; ^3^Department of Neurology, University of California, San Francisco, San Francisco, CA, United States; ^4^Department of Neurological Surgery, University of California, San Francisco, San Francisco, CA, United States; ^5^Department of Epidemiology & Biostatistics, University of California, San Francisco, San Francisco, CA, United States; ^6^Department of Radiation Oncology, University of California, San Francisco, San Francisco, CA, United States

**Keywords:** fMRI, brain connectivity, 7T MRI, radiation therapy, brain tumors, memory, vascular injury

## Abstract

**Background:**

Cognitive impairment and cerebral microbleeds (CMBs) are long-term side-effects of cranial radiation therapy (RT). Previously we showed that memory function is disrupted in young patients and that the rate of cognitive decline correlates with CMB development. However, vascular injury alone cannot explain RT-induced cognitive decline. Here we use resting-state functional MRI (rsfMRI) to further investigate the complex mechanisms underlying memory impairment after RT.

**Methods:**

Nineteen young patients previously treated with or without focal or whole-brain RT for a brain tumor underwent cognitive testing followed by 7T rsfMRI and susceptibility-weighted imaging for CMB detection. Global brain modularity and efficiency, and rsfMRI signal variability within the dorsal attention, salience, and frontoparietal networks were computed. We evaluated whether MR metrics could distinguish age- and sex-matched controls (*N* = 19) from patients and differentiate patients based on RT exposure and aggressiveness. We also related MR metrics with memory performance, CMB burden, and risk factors for cognitive decline after RT.

**Results:**

Compared to controls, patients exhibited widespread hyperconnectivity, similar modularity, and significantly increased efficiency (*p* < 0.001) and network variability (*p* < 0.001). The most abnormal values were detected in patients treated with high dose whole-brain RT, having supratentorial tumors, and who did not undergo RT but had hydrocephalus. MR metrics and memory performance were correlated (*R* = 0.34–0.53), though MR metrics were more strongly related to risk factors for cognitive worsening and CMB burden with evidence of functional recovery.

**Conclusions:**

MR metrics describing brain connectivity and variability represent promising candidate imaging biomarkers for monitoring of long-term cognitive side-effects after RT.

## Introduction

The long-term effects of brain tumor therapies on neurocognitive function and the development of young patients are well known. In the years following radiation therapy (RT), a key contributor to these side-effects, gradual declines in intelligence quotient have been observed (1–3) with notable impairments in memory and executive function (4–6). Several groups have reported more severe impairments in patients treated with RT at younger ages, higher doses, larger irradiated volumes, and for specific tumor subtypes, based on whether they received proton vs. photon RT (1, 2, 4, 5, 7). The mechanisms underlying neurocognitive decline after RT are not fully understood but are thought to be related to RT-induced white matter necrosis, functional changes in neural networks (8), as well as vascular brain injuries that can be observed on magnetic resonance imaging (MRI) as early as 1-year following RT (4, 6, 9).

Previously we leveraged the enhanced spatial resolution and susceptibility contrast benefits of 7 Tesla (7T) susceptibility-weighted imaging (SWI) to investigate the relationship between RT-induced vascular injury and cognitive performance. Vascular injury, in the form of arterial thinning and tiny hemosiderin brain deposits called cerebral microbleeds (CMBs), worsened over time at the same rate with which verbal memory performance was declining (4, 10). Cross-sectional studies at lower field strengths have also linked RT-induced CMBs to cognition (6), which is not surprising as similar pathology has been shown to be related to the cognitive impairments experienced by dementia patients and even healthy aging adults (11, 12). The finding that irradiated patients without detectable white matter necrosis experience long-term cognitive deficits further emphasizes this link between vascular injury and cognition (8), however, vascular injury alone cannot explain other examples of neurocognitive decline such as in non-irradiated patients, suggesting a functional abnormality. While increased alterations in functional connectivity derived from functional MRI (fMRI) have indeed been associated with poorer neurocognitive performance before and after brain tumor therapy (13), there remains little functional data to explain long-term cognitive outcomes, especially after RT.

Resting-state functional MRI (rsfMRI) is a powerful research tool in clinical neuroscience that can detect network alterations by way of indirectly measuring spontaneous fluctuations in brain activity during rest (14). Within millimeter partitions of the brain, the time-varying amplitude of blood-oxygen metabolism in response to neural activity is recorded, and the spatiotemporal synchronicity of the rsfMRI “blood-oxygenation level dependent” (BOLD) signal between brain areas is typically evaluated to measure connection strength and define brain networks. Prior studies have used rsfMRI to demonstrate, for example, that adult survivors of childhood posterior fossa tumors have hyperconnected frontal brain areas relative to controls (15–17). Similarly, task-based fMRI studies involving neurocognitive testing during image acquisition, have revealed long-term functional differences in brain tumor patients as a result of treatment (17) and exposure to cognitive rehabilitative interventions (16, 18). The ability of fMRI to probe cognition (19–21) and predict neurocognitive outcomes in diverse patient groups (22–25) *via* the detection of network alterations, makes it especially useful for investigating the complex mechanisms underlying RT-induced neurocognitive decline. In this study, we acquired rsfMRI and SWI at 7T in a cohort of 19 patients previously treated for a brain tumor in order to relate measures of functional brain network organization and activity to memory performance and vascular injury alongside known clinical risk factors for cognitive decline after RT. Here 7T MRI was used strategically to achieve increased image spatial resolution, enhanced tissue susceptibility and BOLD signal contrast, and ultimately gains in statistical power (26, 27). We also performed a small reproducibility study to investigate the stability of our functional imaging metrics with different preprocessing and analysis parameters, given their known influence on fMRI reliability.

## Methods

### Participants

With institutional review board approval and parental or patient written informed consent, 19 patients (mean age 18 years, range 12–25 years; 47% female) previously treated for a brain tumor underwent 7T rsfMRI, SWI and T1-weighted imaging [see Table 1 for demographics and our prior work (4) for further details on the cohort and recruitment criteria]. Compared to 1.5 or 3T MRI, 7T imaging is associated with heightened risk of claustrophobia and more pronounced patient bioeffects such as dizziness due to the increased field strength, nonetheless the technique remains safe and was well-tolerated by the present cohort. Recruited patients were either non-irradiated or received RT at least 1 month prior to the MRI. Of the 19 patients, eight were treated with whole-brain RT (WBRT) for a medulloblastoma in the posterior fossa, except for one patient who had a pineal parenchymal tumor in the same location. Five patients were treated with whole-ventricular focal RT (WVRT) for a ventricular germinoma; two others were treated with supratentorial focal RT for gliomas located in the occipital and parietal lobe. The remaining four control patients were treated with surgery but not RT for lower-grade gliomas primarily in the posterior fossa. On average, the WBRT group (mean age 19.1 years, range 14–25) was treated 9.8 years prior (range 3.1–19.9); the focal group (including WVRT; mean age 20.1 years, range 14–25) 4.3 years prior (0.1–13); and non-irradiated controls (mean age 15 years, range 13–18) 3.7 years prior (range 0.8–8 years). While several patients had a history of hydrocephalus, a common side-effect of brain tumors linked to cognitive dysfunction (28), only two patients (#13 and #19 in Table 1) showed evidence of ventricle enlargement on MRI. A random selection of 19 age- and sex-matched healthy control data (approx. mean age 19 years, range 11–25; 47% female) from two publicly available 3T rsfMRI datasets (29, 30) were also evaluated in this study for comparison with the patient data (full datasets can be accessed *via* openneuro.org using data accession numbers ds000221 and ds000256).

**Table 1:** Patient demographics.

**Patient**	**Sex**	**Race**	**Cancer Type**	**Tumorlocation**	**RT**	**Age (years)**		**RT dose, max (Gy)**	**Surgery**	**Chemo**	**Other Dx**
						**RT**	**1^st^ MRI**				
1	M	Hispanic	Medu	p. fossa	wb	3	22	37, 53.5	gtr ×4	Vincristine	–
2	F	White	Medu	p. fossa	wb	18	22	36, 55.8	biopsy	Cisplatin, cyclophosphamide, vincristine	–
3	M	White	Medu	p. fossa	wb	14	22	23.5, 54	gtr	Carboplatin, CCNU, cytoxan, VP-16, vincristine	–
4	M	White	Medu	p. fossa	wb	6	25	36, 55	gtr	Carboplatin, CCNU, vincristine	Hypertension
5	M	White	Medu	p. fossa	wb	7	22	23.4, 55.8	gtr	CCNU, cisplatin, vincristine	–
6	M	White	Medu	p. fossa	wb	12	14	36, 54	str	Carboplatin, vincristine	–
7	F	White	Medu	p. fossa	wb	9	14	23.4, 54	gtr	Carboplatin, CCNU, cisplatin, cyclophosphamide, cytoxan, vincristine	–
8	M	Asian	Germ	Ventricle	wv	9	19	24, 45	ETV, biopsy	Carboplatin, VP-16	Diabetes, hydro cephalus
9	M	White	Germ	Ventricle	wv	22	22	18, 30	str	Carboplatin, etopside, ifosfamide	Diabetes
10	F	White	Germ	Ventricle	wv	9	22	24, 40.5	biopsy	Carboplatin, VP-16	Diabetes
11	F	Other	Germ	Ventricle	wv	12	14	18, 30	biopsy	Carboplatin, VP-16	Diabetes
12	F	Asian	Germ	Ventricle	wv	24	25	18, 33	biopsy	Carboplatin, VP-16	–
13*	M	White	PPT	p. fossa	wb	9	12	23.4, 54.9	gtr, ETV	Cisplatin, cyclophosphamide	Hydro cephalus, stroke
14	M	White	gAnglio	Occipital	focal	15	17	59.4, 59.4	gtr	Vemurafenib	–
15	M	Black	Astro	Parietal	focal	22	22	59.4, 59.4	gtr	–	–
16	F	White	OLIGO	Temporal	–	–	15	–	gtr	Everolimus	–
17	F	White	JPA	p. fossa	–	–	18	–	gtr	–	–
18	F	White	JPA	p. fossa	–	–	13	–	gtr	–	Hydro cephalus
19	F	Hispanic	JPA	p. fossa	–	–	14	–	ETV, biopsy	–	Hydro cephalus

medu, medulloblastoma; germ, germinoma; PPT, pineal parenchymal; ganglio, anaplastic ganglioglioma; astro, pleomorphic xanthoastrocytoma; oligo, oligodendroglioma; JPA, juvenile pilocytic astrocytoma; p.fossa, posterior fossa; wb, whole brain; wv, whole ventricular; n/a, data affected by motion; gtr, gross total resection; str, sub-total resection; ETV, endoscopic third ventriculostomy.

### Neurocognitive testing

Prior to the MRI exam, a battery of seven computerized cognitive tests (Cogstate, Inc.; Newhaven, CT) were administered (4). In a previous analysis of this cohort we found the verbal memory test involving recall of items on a shopping list (International Shopping List; ISL) to be most useful for distinguishing patients who were treated with vs. without RT (4). We therefore focused the present work on the ISL test and corresponding brain networks involved in episodic memory (31, 32). The total number of correct items recalled was converted into an age normalized z-score based on mean test scores of healthy controls from Cogstate's database.

### Imaging protocols

Imaging was performed on a 7T General Electric (GE) Healthcare scanner equipped with a 2-channel transmit and 32-channel receive head coil. Resting-state fMRI scans were acquired using an interleaved, gradient-echo sequence with 125 time points [repetition time (TR) = 4 s, minimum echo time (TE), flip angle = 90, 1.8 mm isotropic resolution, 23 cm field-of-view (FOV)]. TRs for the public fMRI acquired at clinical field-strengths were 1.4 s (29) and 2.5 s (30). SWI and T1-weighted images were also acquired; key MR parameters for these sequences are included in Morrison et al. (4), with a more in-depth description of the simultaneous MRA-SWI sequence and reconstruction methods provided in Bian et al. (33).

### Data analysis

We investigated aspects of brain network organization and activity from the rsfMRI data by computing: (1) theoretical graph metrics representing brain network modularity and efficiency and (2) measures of BOLD variability thought to represent the brain's cognitive flexibility, namely its ability to efficiently process and respond to unexpected external stimuli (34). These metrics were chosen as modularity has previously been shown to predict the efficacy of cognitive rehabilitative interventions in young adults (35), while efficiency appears to mediate risk for vascular injury and the development of cognitive impairments (36) and is linked to cognitive flexibility in pediatric brain tumor survivors (37).

#### fMRI preprocessing

**Figure 1:**
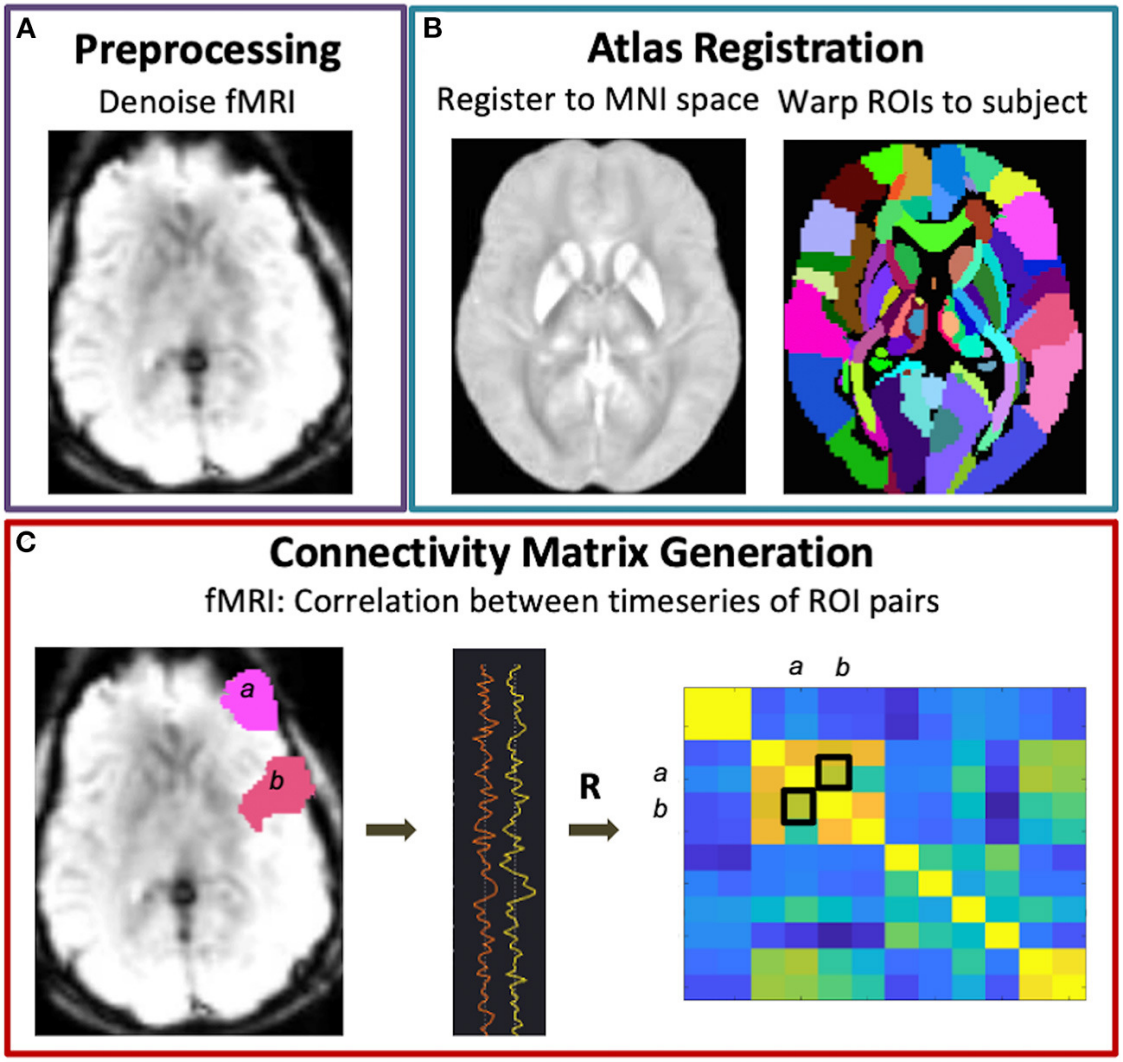
Functional connectivity matrix generation. Following fMRI data preprocessing **(A)** and warping of the brain atlas regions-of-interest (ROIs) to subject space **(B)**, connectivity matrices were generated *via* Pearson correlations between all pairs of brain ROIs **(C)**.

Preprocessing was performed using the default pipeline in CONN (38). Steps included motion estimation and realignment correction, slice-timing correction, outlier detection, segmentation of the brain tissue, registration of the data to an atlas space (Montreal Neurological Institute (MNI) brain atlas), and spatial smoothing with an 8mm Gaussian kernel (Figures 1A,B). Bandpass filtering (0.01–0.25 Hz) and linear regression thereafter removed the effects of confounding covariates including outliers based on >2 mm translation (*N*_max_ = 30), motion parameters (*N* = 12), and noise components in regions dominated by the white matter (*N* = 15) and cerebrospinal fluid (*N* = 5). The same denoising parameters were used for all subjects but adjusted such that each subject's whole brain connectivity values were normally distributed after denoising while maintaining minimum 30 degrees of freedom.

#### Network modularity and efficiency

Graph metric analysis was performed using CONN (38) and the Brain Connectivity Toolbox (BTC) (39). For each subject, an adjacency matrix representing nondirected, whole brain functional connectivity was generated by correlating the mean BOLD signal across pairs of brain regions derived from 132 atlas parcellations (Figure 1C). In this way, the nodes of the graph represent the brain parcels, while the edges correspond to connections. A threshold of *r* > 0.5 was applied to the subject-level matrices such that only positive, moderate-to-strong connections were used to compute global modularity and efficiency. Modularity is a measure of the degree to which the network is divided into smaller, nonoverlapping subgroups such that within-group edges are maximized while between-group edges are minimized, and was computed as


(1) $$\begin{array}{r} \overset{N}{\underset{i=1}{\sum }}\left(e_{ii}-a_{i}^{2}\right) \end{array}$$

where *N* is total number of subgroups, e_*i*_ is the proportion of edges connecting any two nodes within subgroup *i*, and *a*_*i*_is the proportion of edges connecting an individual node in subgroup *i* to any other nodes including nodes in other subgroups (40). Global efficiency is an inverse measure of the average shortest path length (or smallest number of edges) between all pairs of nodes, and was computed as


(2) $$\begin{array}{r} \frac{\text{Σ}\left(di\left(:\right)\right)}{\left(n^{2}-n\right)} \end{array}$$

where *di* is the inverse shortest distance between nodes, and *n* is the total number of nodes (41).

#### Network variability

BOLD variability was computed as the standard deviation of time series data corresponding to three networks of interest, isolated *via* independent component analysis (ICA) decomposition in CONN (40 components with 64 component subject-level dimensionality reduction). These networks included the salience, frontoparietal, and dorsal attention networks that are commonly engaged during working memory processes based on a meta-analysis search in neurosynth.org of 1,091 imaging studies. The networks were identified using a spatial matching template and if left and right network activity appeared as two separate components, both components were used in the analysis. To verify that our cohort of patients had altered functional connectivity involving nodes of these networks, we performed an atlas- and voxel- based *t*-test comparing connectivity maps for patient vs. control using seed-based correlations to generate the maps for each network. Here the mask of the entire atlas-defined network was used as the seed. Since ICA decomposition can return different solutions based on the choice of preprocessing and analysis parameters, we also performed a small reproducibility study using data from the present cohort and a separate cohort of adult patients with Parkinson's disease imaged at 3T to evaluate the effects of bandpass filtering (0.008–0.09 Hz vs. 0.008–0.06 Hz vs. 0.016–0.09Hz), spatial smoothing (none vs. 4 vs. 8 mm) and number of ICA components (20 vs. 40, where 20 is the minimum to extract a complete set of resting-state networks) on the variability of the dorsal attention network (DAN).

#### Statistical analysis

We used Wilcoxon rank sum tests to determine whether MR metrics could distinguish healthy controls from patients and amongst patient subgroups: no RT, focal RT (including supratentorial focal and whole ventricular focal RT), and WBRT (including low and high dose whole brain RT). Pearson correlation coefficients were estimated for relationships between age-normalized MR metrics and ISL performance scores, as well as total CMB burden. We used linear regression to test whether known risk factors for RT-induced neurocognitive impairment, such as age during RT and time since RT, were related to ISL performance scores and MR metrics to further evaluate the potential of modularity, efficiency, and BOLD variability as markers of cognition.

## Results

### Reliability of network variability metric

**Figure 2:**
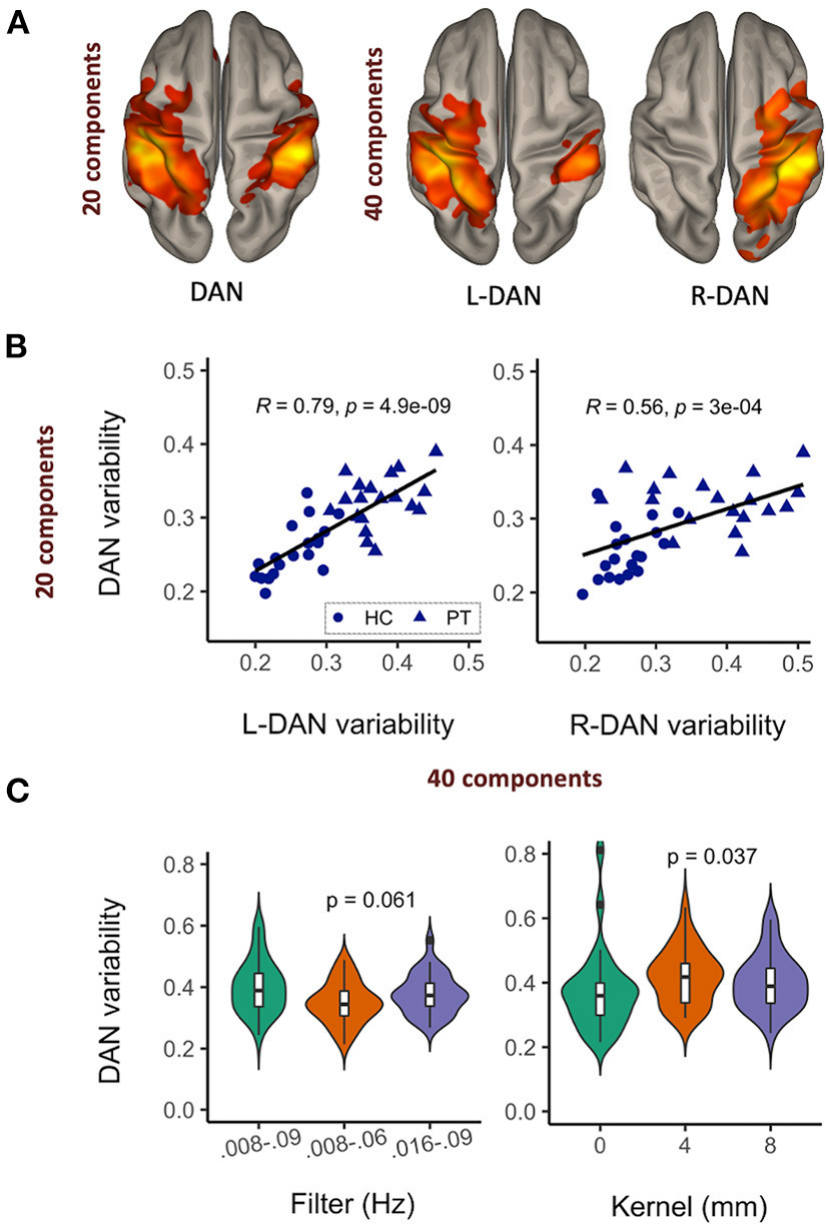
Network variability is a reproducible and stable MR metric. Upon increasing the number of ICA components, the dorsal attention network (DAN) was reduced to left and right hemisphere sub-networks **(A)**, with corresponding variability metrics remaining significantly correlated **(B)** and relatively stable across different data filtering bandwidths and spatial smoothing kernels **(C)**.

The results of our reproducibility study are shown in Figure 2. Upon increasing the number of ICA components from 20 to 40, the DAN was reduced into left and right hemisphere subnetworks (Figure 2A). Network variability computed from the DAN time courses corresponding to 20- vs. 40-component ICA were significantly correlated (Figure 2B). The left hemisphere subnetwork was more strongly correlated with the overall DAN variability than the right subnetwork (L-DAN_40_ vs. DAN_20_: *R* = 0.79, *p* < 0.0001; R-DAN_40_ vs. DAN_20_: *R* = 0.56, *p* < 0.0001), while healthy control data appeared to be more tightly correlated than patient data. Though not significant, the use of a narrower and lower frequency bandwidth i.e., 0.008–0.06 Hz yielded lower variability than the use of a wider bandwidth, i.e., 0.008–0.09 Hz (Figure 2C). Unsmoothed data also produced lower variability values on average, a finding that was significant when compared to standard 4mm and 8mm kernel smoothing (Figure 2C). Nonetheless, the overall effect size was small relative to the range of values typically measured across healthy controls and patients (see Figure 2B). Taken together, these results reiterate that the BOLD variability metric derived from ICA-based network time series data is reproducible and stable across different preprocessing and ICA parameters.

### Distinguishing types of RT exposure and aggressiveness with rsfMRI metrics

**Figure 3:**
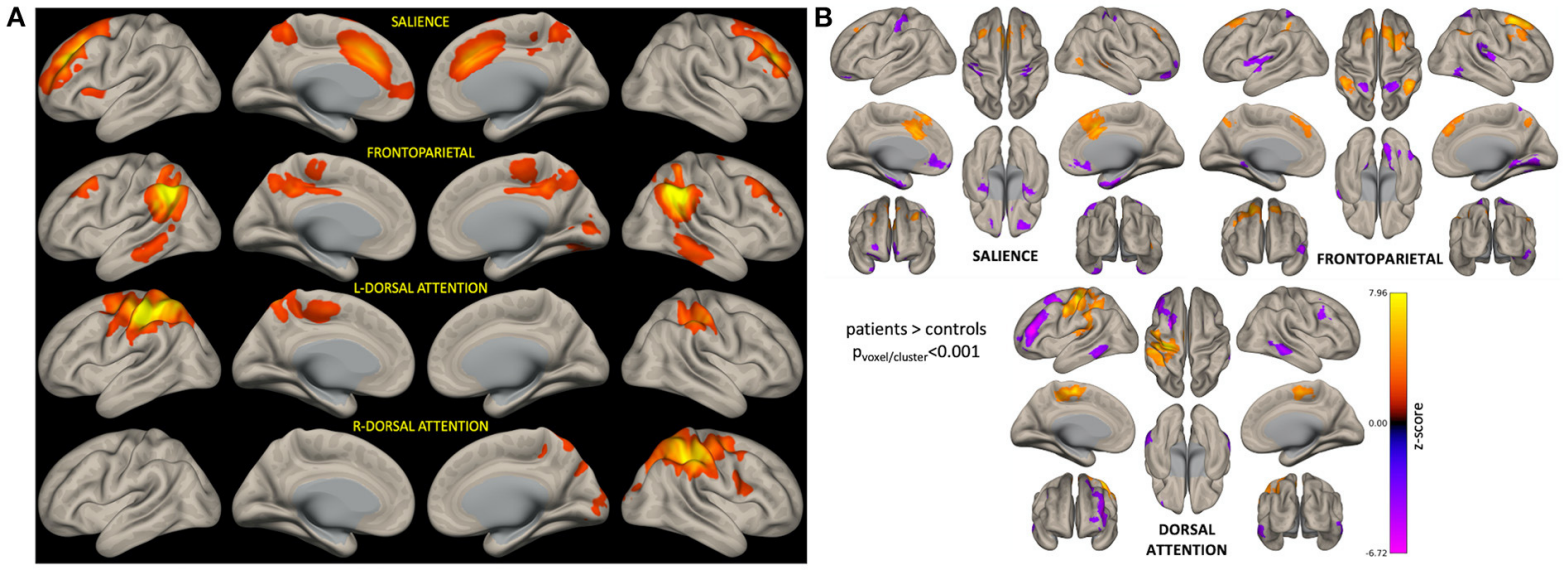
ICA-derived brain networks for all subjects and seed-based analysis confirming altered networks connectivity in patients. For each patient, BOLD variability was computed as the standard deviation of network-specific timeseries data **(A)**. Seed-based analysis confirmed that patients had significantly altered connectivity (relative to controls) involving nodes of the networks of interest **(B)**. In **(B)**, orange corresponds to brain areas that are hyperconnected to respective network nodes in patients (relative to controls), while purple represents hypoconnected areas.

**Figure 4:**
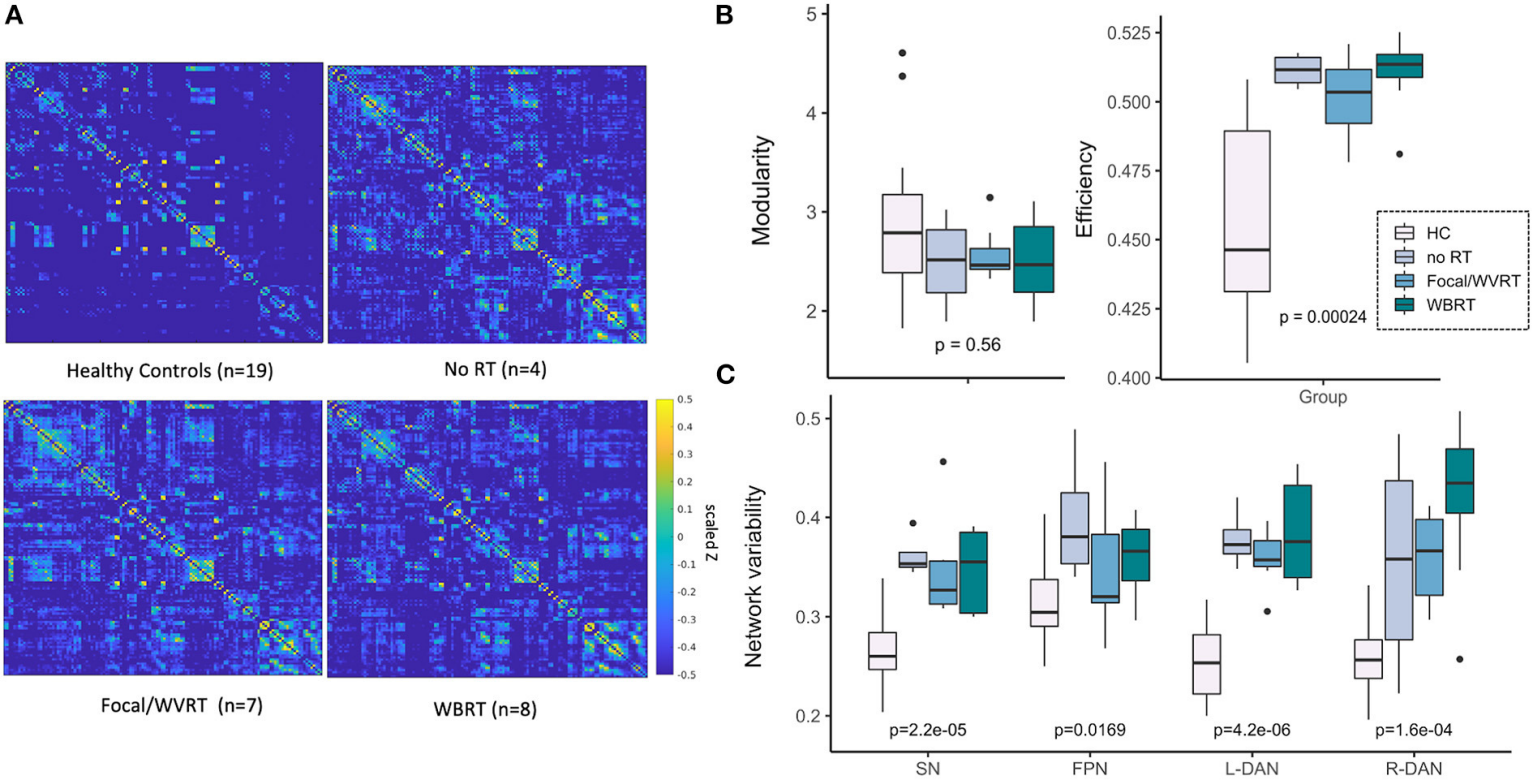
MR metrics can distinguish healthy controls and patient subgroups. Scaled and Fisher transformed functional connectivity (FC) matrices reveal notable group differences in global connectivity **(A)**. MR metrics derived from FC matrices and ICA brain networks show significant group trends related to group exposure to radiation therapy (RT) and/or the degree of RT treatment aggressiveness **(B, C)**. HC, healthy controls; no RT, non-irradiated patients; WVRT, whole-ventricular focal RT; Focal, focal RT to the supratentorial brain; WBRT, whole-brain RT.

Figure 3A shows brain networks of interest averaged across patients and controls, from which network variability was computed. Based on a 40-component ICA, a total of four networks were identified including the left and right hemisphere DAN, salience network (SN), and frontoparietal network (FPN). Individual subject networks constructed *via* backprojection spatially reflected the total group average; tumor sites did not overlap with the networks. Comparison of network connectivity for patients vs. controls using seed-based correlations confirmed altered patient connectivity, including hyperconnectivity in medial frontal nodes of the SN and FPN, and parietal nodes of the FPN and DAN (Figure 3B). Reduced connectivity was also observed between lateral frontal areas involved in memory and language, and the DAN and FPN. Qualitative evaluation of group-averaged functional connectivity matrices, from which global efficiency and modularity were computed, revealed noticeable global connectivity differences (Figure 4A). Compared to the healthy controls (*n* = 19), all patients had more widespread hyperconnectivity. Upon separating patients into finer group (Supplementary Figure 1), those treated with focal RT to the supratentorial brain (*n* = 2) exhibited extensive hyperconnectivity, followed by those treated with high-dose WBRT (*n* = 4), no RT (*n* = 4), low-dose WBRT (*n* = 4), and WVRT (*n* = 5).

All imaging metrics except network modularity showed significant group differences (Figures 4B,C). We expected to observe trends in imaging metrics across the groups according to whether RT was received, followed by treatment aggressiveness based on the approximate volume, location, and dose of RT. In this way and to maintain sufficiently sized groups, we first ranked the groups as follows: healthy controls < no RT < focal/WVRT RT < WBRT. In a supplementary analysis we investigated finer groupings: healthy controls < no RT < WVRT < supratentorial focal < low-dose WBRT < high-dose WBRT. As seen in Figures 4B,C, global efficiency and local SN and DAN variability best reflected this expected trend where group-level RT exposure and increasing treatment aggressiveness appeared to be associated with higher global efficiency (*p* < 0.001) and SN and DAN variability (all *p* < 0.001). While healthy controls nearly consistently yielded significantly lower values than patients, high values were unexpectedly recorded for non-irradiated patients, often exceeding that of the focal RT patients. Similar plots in Supplementary Figure 1 with patients separated into finer groups, showed the same trends plus a consistent effect of dose whereby high-dose WBRT was associated with higher MR metrics.

### rsMRI metrics and memory performance

**Figure 5:**
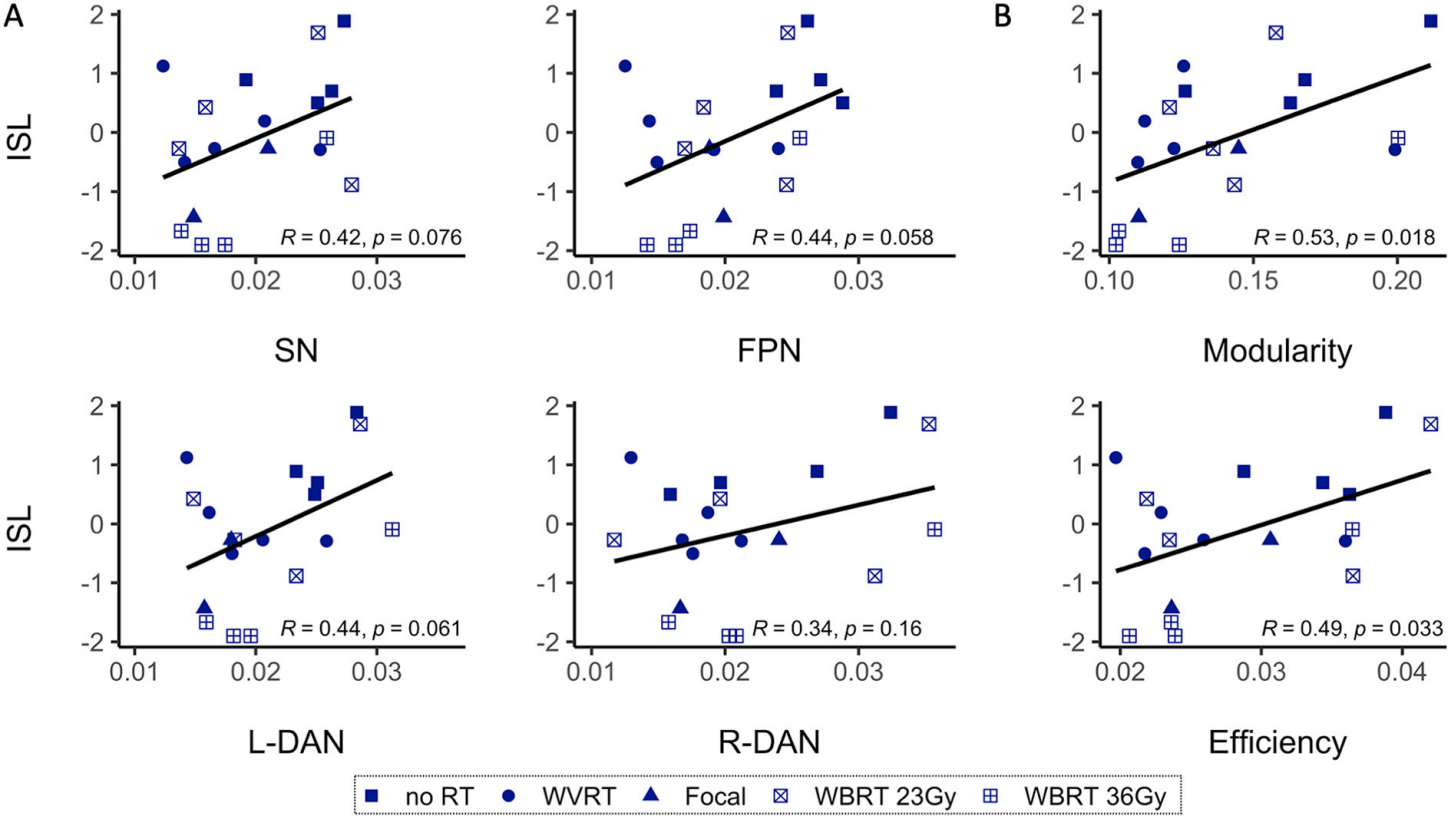
Age-normalized MR metrics and ISL test scores are correlated. Age-normalized network variability **(A)** and functional connectivity **(B)** metrics show a positive correlative trend with the age-normalized International Shopping List (ISL) test scores, representative of neurocognitive memory performance. As shown in B, only modularity and efficiency are significantly correlated with ISL. SN, salience network; FPN, frontoparietal network; L-DAN, left hemisphere dorsal attention network; R-DAN, right hemisphere DAN.

Correlation tests between patients' age-normalized MR metrics and the ISL test scores, representative of memory performance, revealed a significant positive correlative relationship between global modularity and efficiency and ISL scores (modularity: *R* = 0.53, *p* < 0.02; efficiency: *R* = 0.49, *p* < 0.05; Figure 5B). Network variability metrics similarly showed near significant, positive correlative trends with ISL scores except for R-DAN variability (Figure 5A). Division of the MR metrics by age at the time of imaging to produce age-normalized values, limits direct interpretation of trend lines in Figure 5 where performance decline appears to be associated with lower MR values. Nonetheless, age-normalization here was necessary as ISL test scores are normalized to age-appropriate healthy control data, and furthermore functional connectivity and network variability metrics have shown age-related changes across the lifespan (20).

### Relationship between CMB burden and rsfMRI metrics and memory performance

**Figure 6:**
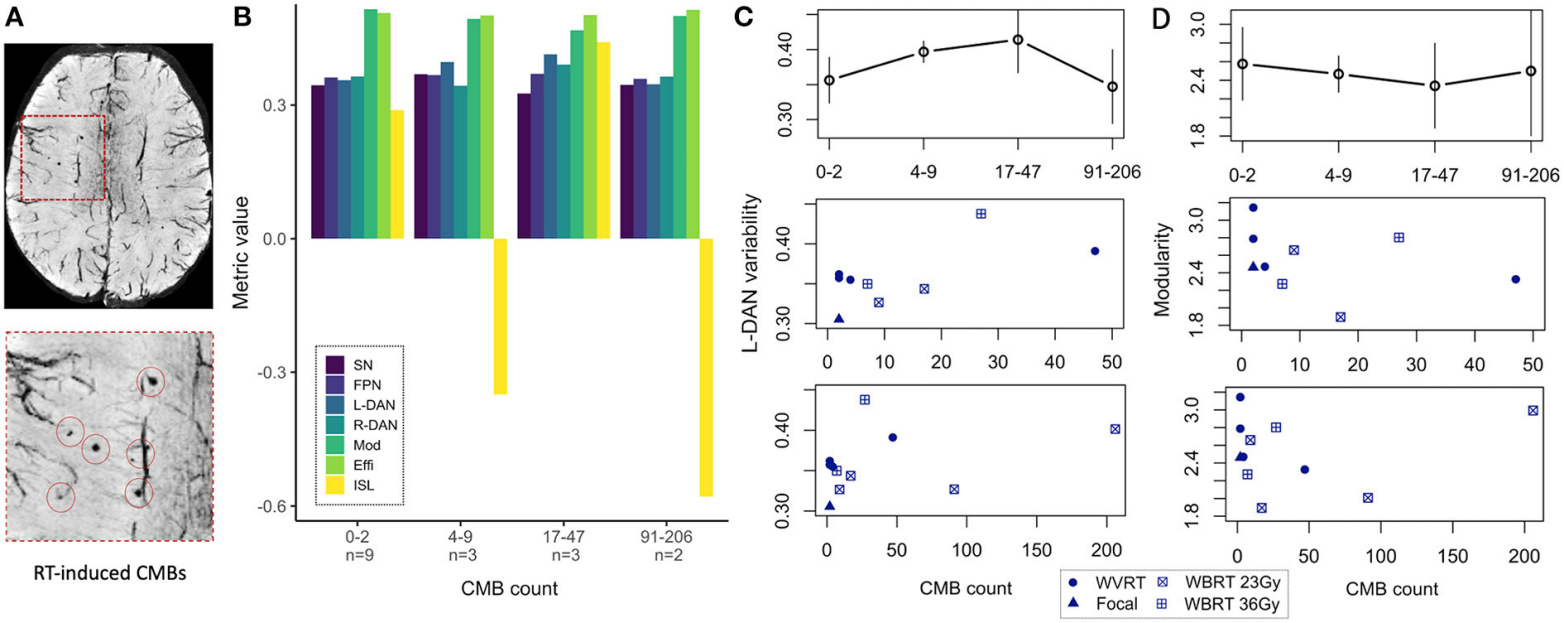
MR metrics change parabolically with increasing CMB burden. Degree of cerebral microbleed (CMB) burden detected on SWI **(A)** reveals parabolic-like changes in left hemisphere dorsal attention network (L-DAN) variability and modularity **(B)** with increasing CMB burden (based on within-group averages). Isolated plots for L-DAN variability and modularity are shown respectively in the top row of **(C, D)**; the bottom rows show the individual patient data points for CMB counts ranging 1–50 (magnified) and 1–205 (full range). In **(B)**, modularity values have been reduced by a factor of 5 for visualization purposes. Two of the 19 patients had poor SWI data quality and therefore CMB burden could not be evaluated. SN, salience network; FPN, frontoparietal network; L-DAN, left hemisphere dorsal attention network; R-DAN, right hemisphere DAN; Mod, modularity; Effi, efficiency; ISL, International Shopping List test score.

In our prior work (4), CMB burden defined as the total number of CMBs detected on a patient's SWI images (Figure 6A), showed only longitudinal correlations with neurocognitive performance and here our results reiterate that cross-sectional ISL test scores do not show any clear trend with CMB burden (Figure 6B, *yellow bars*). Our approach of regrouping patients by their CMB burden based on the distribution of the data allowed for the inclusion of patients with no CMBs and revealed an interesting trend for L-DAN variability and modularity. Notably, L-DAN variability appeared to follow a parabolic trend where patients with low (0–2 CMBs) and very high CMB burden (>91 CMBs) averaged low variability, while patients with moderate CMB counts (4–47) averaged high variability (Figure 6C, top row). The opposite was observed for modularity where high modularity was detected in patients with very low and very high CMB counts, while low modularity was found in patients with moderate CMB counts (Figure 6D, top row). Plotting of the individual patient data points for those who had at least 1 CMB (Figures 6C,D, bottom rows) showed that the results are largely dependent on imaging values from the few subjects with >100 CMBs. These parabolic-like trends for L-DAN variability and modularity can also be mildly appreciated in Figures 4B,C, despite grouping patients based on RT exposure and aggressiveness as opposed to CMB burden.

### Association of risk factors for cognitive decline after RT with rsfMRI metrics and memory performance

**Figure 7:**
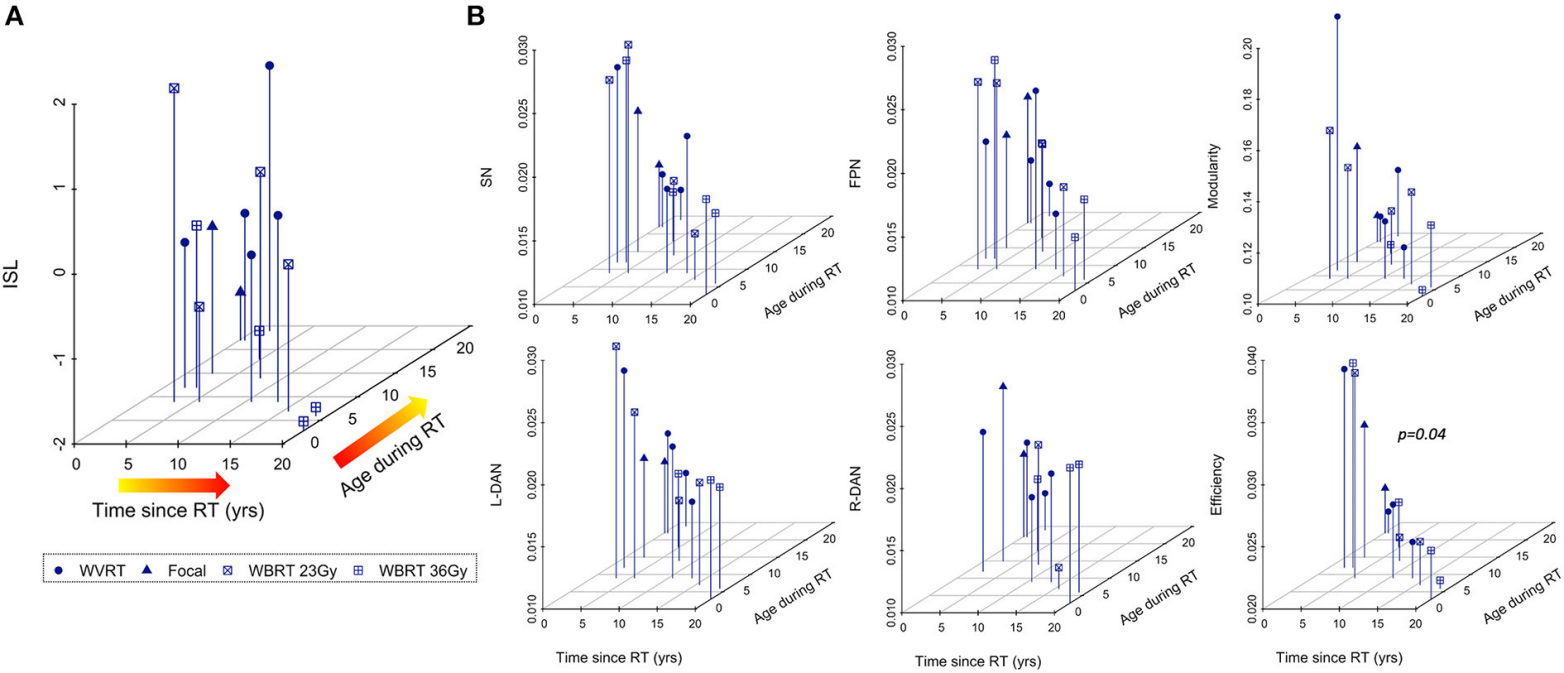
Age-normalized MR metrics are strongly associated with risk factors for cognitive decline after RT. Neurocognitive memory performance measured *via* the international shopping list (ISL) task is less associated with known risk factors for neurocognitive decline after RT **(A)** than MR metrics **(B)** representing surrogate markers of neurocognitive status.

Younger age during RT and increased time elapsed since RT are known risk factors for neurocognitive decline and CMB development after radiation exposure (1, 3, 4, 9). Multiple regression analysis revealed significant associations between age-normalized imaging metrics and risk factors for neurocognitive decline, with global efficiency yielding the strongest association followed by SN variability; ISL test scores were not significantly associated with the risk factors (Table 2). Although these results are illustrated in Figure 7 as three-dimensional scatter plots, normalization of the MR metrics by age at the time of imaging again limits the direct interpretation of these trends.

**Table 2:** Multivariate analysis of risks factors for neurocognitive decline after RT.

**Neurocognitive outcome metric^a^**	**Age during RT**		**Time since RT**	
	**Incidence rate ratio [95% confidence interval]**	***p*** **-Value**	**Incidence rate ratio [95% confidence interval]**	***p*** **-Value**
**Neurocognitive performance:**
International shopping list	−0.06 [−0.20, 0.08]	NS	−0.11 [−0.23, 0.02]	NS
**Network variability:**
Salience	−0.001 [−0.0015, −0.0009]	<0.0001	−0.001 [−0.0014, −0.0008]	<0.0001
Frontoparietal	−0.0008	<0.002	−0.0008	<0.002
L-dorsal attention	−0.001 [−0.0015, −0.0007]	<0.0001	−0.001 [−0.0014, −0.0006]	<0.002
R-dorsal attention	−0.0015 [−0.0022, −0.0008]	<0.002	−0.0013 [−0.0020, −0.0007]	<0.002
**Functional connectivity:**
Modularity	−0.0055 [−0.0087, −0.0023]	<0.005	−0.0057 [−0.0087, −0.0023]	<0.002
Efficiency	−0.0017 [−0.0019, −0.0014]	<0.0001	−0.0016 [−0.0018, −0.0014]	<0.0001

a Imaging metrics represent surrogate measures of neurocognitive status and are normalized by age at the time of imaging.

## Discussion

This cross-sectional imaging study investigated how rsfMRI measures of brain connectivity and network variability differ among patients with varying exposure to RT and degree of treatment aggressiveness compared to age- and sex-matched healthy controls. The strength of functional brain connections as well as variability within certain networks was related to: (1) neurocognitive measures of memory performance, (2) vascular injury in the form of CMBs, and (3) known risk factors for neurocognitive decline after RT, demonstrating that functional MR metrics could be useful surrogate markers of cognition in brain tumor patients for reliable evaluation of the long-term treatment side-effects.

Reliability of the BOLD signal in patients with brain tumors has previously been shown to vary with disease aggressiveness (42). Since BOLD variability has not yet to our knowledge been investigated as a potential biomarker of brain tumor features or treatment side-effects, the first aim of this study was to demonstrate its reproducibility. Overall, we found that BOLD variability metrics were relatively stable across fMRI preprocessing and ICA postprocessing parameters, though unsmoothed data yielded several outliers. Spatial smoothing is generally considered an essential preprocessing step to improve data signal-to-noise ratio, with previous studies having shown stable functional connectivity as a function of resolution after smoothing (43). However, other studies have argued that spatial smoothing should be avoided in network analyses (44). While the results cannot inform the optimal approach, they do show that BOLD variability is more reproducible among different smoothing kernels, and that unsmoothed data significantly reduces the relative group average.

By nature of the ICA decomposition method, fMRI data-driven outputs (spatial brain maps and associated time courses) vary with each iteration (45). The desired number of components typically chosen in line with the experimental hypothesis can also significantly alter the solution, yielding brain maps of (sub)-networks of different granularity [as shown in Figure 2A and thoroughly explored by Wang and Li (46)]. Our finding that 20 and 40 component ICA produces BOLD variability metrics that are considerably correlated in patients, provides impetus for proceeding to use these metrics for biomarker discovery, and demonstrates that while ICA solutions can appear spatially distinct with increased number of components, the time courses can look relatively similar. Compared to the patient data, the control subject data were even more tightly correlated despite being derived from two different public datasets, providing further evidence that the BOLD variability metric is also robust against scanner and acquisition parameters.

Independent component analysis with 40 components (twice the minimum to extract a complete set of resting-state networks) was used to resolve more fine-grained representations of the network that may better characterize the underlying neurophysiological complexity of working memory processes. Seed-based analysis detected significant differences in SN, FPN, and DAN connectivity between patients and controls, providing further rationale for their inclusion as networks of interest to compute BOLD variability. Specifically, the finding of frontal lobe hyperconnectivity in patients agrees with previous reports of survivors of childhood tumors exhibiting frontal hyperconnectivity in the FPN and SN relative to controls, (15) as well as increased frontal engagement during working memory tasks (47). In young patients this is not surprising as frontal regions have been shown to develop post-adolescence and thus remain highly susceptible to plasticity (48).

On average, with the exception of global brain modularity, patients exhibited higher functional connectivity metrics than age- and sex-matched controls, providing further evidence of functional alterations in patients associated with treatment related side-effects despite the likely influence of previous structural changes due to lesion growth. Increased BOLD variability has previously been detected linearly across the lifespan and associated with age-related reductions in cognitive performance (49–51). In adults with Alzheimer's disease, Scarapicchia and colleagues also found that during rest whole-brain BOLD variability was increased in patients relative to controls and furthermore related to lower memory scores (52). Functional imaging studies of network efficiency have similarly shown enhanced efficiency in patients relative to controls (53). Given that both these metrics capture information about cognitive flexibility, their elevation in patients with increasing RT exposure and aggressiveness in this study could reflect underlying compensatory neural mechanisms directly related to the severity of their experienced brain injury. It is also important to note that while structural network efficiency reflecting impaired white matter architecture is often *decreased* in patients (36, 37), fMRI metrics are uniquely advantageous in that they can capture polysynaptic activity and early neuroplastic changes that might not be reflected structurally. Although brain modularity was not as sensitive to patient vs. control differences, controls on average exhibited slightly higher modularity which aligns with prior evidence of young individuals with higher baseline modularity performing better over iterative cognitive training sessions (35).

Qualitative evaluation of the group averaged functional connectivity matrices and the inclusion of age- and sex-matched controls in addition to non-irradiated controls was especially critical to realizing group trends in the data involving RT exposure and aggressiveness. Interestingly, no RT visually exhibited more hyperconnectivity than patients treated with more aggressive high and low dose WBRT regimens for a posterior fossa tumor, respectively. This highlights concerns that non-irradiated patients may not always be suitable controls for evaluating the impact of RT on brain connectivity, and that structural changes due to supratentorial lesion growth or hydrocephalus (detected in 50% of nonirradiated patients) (54) may have a greater impact on functional connectivity than RT effects. Patients treated with focal WVRT had the most normal appearing connectivity profiles, which could be explained by the location and relatively small size of their pre-treatment lesions, as well as the fact that some of the patients were treated as young adults which has previously been associated with a better prognosis (4). Quantitatively, some of the rsfMRI metrics mimicked these visual trends, but we also observed instances in which patients treated with focal RT had more favorable functional connectivity metrics than the WBRT group. While individual patient variations likely influenced differences in the group trends observed across connectivity metrics, the effect of dose within the WBRT group was one trend that remained consistent, providing strong evidence that higher whole-brain doses lead to more functional brain alterations.

Positive correlations observed between the ISL test scores and rsfMRI metrics reaffirm that functional imaging can indeed probe cognition in pediatric brain tumor patients. From the results in Figure 6 relating ISL scores and functional connectivity metrics with CMB burden, we can appreciate the added value of rsfMRI metrics which allowed for meaningful trends in the data to be extracted that otherwise could not be explained by memory task performance alone. Parabolic trends in modularity and DAN variability suggest that there may be a process of functional neural recovery unfolding over the course of years, simultaneous to the development of CMBs over time (4, 6, 9). In this way, one might reconsider the relative influence that vascular injury vs. microstructural and network-level functional changes have on the cognitive abilities of these patients. While severe vascular brain injury can independently lead to functional reorganization, there is evidence for example of re-emergence of modular brain networks in stroke patients that are driven by changes in brain connectivity (55). Given this knowledge, it is not surprising that patients in this study with upward of 100 CMBs (who are also at high risk for stroke), approached normal modularity values. Biologically, modular networks have been proven more functionally efficient than non-modular networks (56), therefore, it also makes sense that we see a re-emergence of cognitive flexibility in the DAN as it relates to the brain's ability to efficiently process and respond to unexpected external stimuli (34).

Further emphasizing the utility of rsfMRI for the evaluation of cognitive side-effects after RT was the finding that risk factors (i.e., time since RT, age during RT) were more strongly associated with rsfMRI metrics than ISL test scores. Although cognitive testing batteries represent the gold standard for detecting cognitive impairments, their cross-sectional reproducibility in young patients is limited (57). Individual performance on a given task can fluctuate with testing fatigue during the exam and even the time of day (58). While rsfMRI metrics have their own limitations with respect to reproducibility (59), our results demonstrate that despite sources of variation influencing reliability, consistent and clinically meaningful trends in the data can still be elucidated using standard preprocessing methods.

There are several limitations to this study, with the most notable ones being the limited cohort size and patient heterogeneity with respect to age, tumor location and pathology, the presence of hydrocephalus, treatment strategy, and time since treatment. We used 7T MRI to enhance statistical power, and to minimize within-group variations and allow for meaningful results to be derived from the limited cohort, we grouped patients based on the similarity of their treatment strategy and naturally the alikeness of their tumor type and location. Nonetheless, factors such as hydrocephalus, although only mildly present in few patients across the groups, may have confounded the results given known independent effects of hydrocephalus on white matter structure and cognition (54). Especially in the non-irradiated control groups where two out of four patients presented with hydrocephalus, we expect that this side-effect significantly influenced the observed connectivity patterns.

Much of these limitations are largely due to unforeseen challenges with recruiting patients for a 7T research scan in addition to their clinical scan, as well as a delay in acquiring rsfMRI data as part of the study protocol. Specifically, challenges in recruiting patients who received WBRT for a posterior fossa tumor >1 year prior resulted in a widening of our inclusion criteria leading to the recruitment of two patients treated <1 year before 7T imaging. Consequently, some of the observed memory impairment and functional alterations in these patients may be caused by acute as opposed to late RT effects, which differ in pathophysiology. Otherwise, a delay in acquiring functional data for this study led to our inability to collect sufficient longitudinal rsfMRI data which, based on our prior work (4), may provide better insight into the relationship between risk factors, cognitive decline, and vascular injury. One final limitation worth noting was our inability to access age-appropriate control data acquired at 7T, thus requiring utilization of public 3T data. Variations in image signal-to-noise ratio (SNR) caused by the differing field strengths may have contributed to the distinction of patient and control metrics; nonetheless, given extensive prior evidence of abnormal brain activity in patients with neurological conditions, the underlying biological effects are still thought to be largely influencing the results. Our control dataset arising from two different studies, scanners, and sequence parameters further demonstrate that the imaging metrics are robust against differences in scanning methods that may affect SNR.

Overall, despite these limitations, the results of this work demonstrate that cross-sectional measures of functional brain connectivity and variability derived from rsfMRI may provide surrogate markers of cognition for monitoring the long-term effects of RT, including the complex relationship between vascular injury, network connectivity, and cognition. While we did not investigate the impact of underlying structural connectivity changes and presence of white matter pathologies on patient outcomes in this study, it is the subject of our ongoing work.

## Conclusion

Collectively, the results demonstrate that rsfMRI metrics describing global brain modularity, efficiency, and local network variability hold promise for monitoring the long-term cognitive side-effects of RT in young patients being treated for a brain tumor. These neuroimaging metrics correlated with memory performance and were also able to effectively differentiate patients based on exposure to and aggressiveness of RT. Compared to memory performance, these functional connectivity metrics were more strongly associated with risk factors for cognitive decline and severity of RT-induced vascular injury.

## Data availability statement

The raw data supporting the conclusions of this article will be made available by the authors, without undue reservation.

## Ethics statement

The studies involving human participants were reviewed and approved by University of California San Francisco Institutional Review Board. Written informed consent to participate in this study was provided by the participant or their legal guardian/next of kin.

## Author contributions

JL and SM designed and directed the overarching study. EF and SS performed recruitment and scheduling and cognitive testing. SM and SB supported patient recruitment. AJ performed MRI data collection and transfer. MM conceptualized the methods and approach and conducted the analyses alongside SW who performed the metric reproducibility analysis. AM provided statistics support for the analyses. CH provided neuroradiology support for the analysis of vascular injury. MM prepared the manuscript and figures. All authors discussed the results and provided written feedback on the manuscript.

## Funding

This work was funded by R01HD079568 NIH NICHD grant.

## Conflict of interest

The authors declare that the research was conducted in the absence of any commercial or financial relationships that could be construed as a potential conflict of interest.

## Publisher's note

All claims expressed in this article are solely those of the authors and do not necessarily represent those of their affiliated organizations, or those of the publisher, the editors and the reviewers. Any product that may be evaluated in this article, or claim that may be made by its manufacturer, is not guaranteed or endorsed by the publisher.

## Supplementary material

Supplementary Figure 1 | MR metrics can distinguish healthy controls and patient subgroups. Scaled and Fisher transformed functional connectivity (FC) matrices reveal notable group differences in global connectivity **(A)**. MR metrics derived from FC matrices and ICA brain networks show significant group trends related to group exposure to radiation therapy (RT) and/or the degree of RT treatment aggressiveness **(B, C)**. HC, healthy controls; no RT, non-irradiated patients; WVRT, whole-ventricular focal RT; Focal, focal RT to the supratentorial brain; WBRT, whole-brain RT.
