# Supplementary figures and images for: Functional network alterations in young brain tumor patients with radiotherapy-induced memory impairments and vascular injury
Source: Front Neurol. 2022 Sep 12;13:921984. doi: 10.3389/fneur.2022.921984 (PMC9511024; doi:10.3389/fneur.2022.921984)

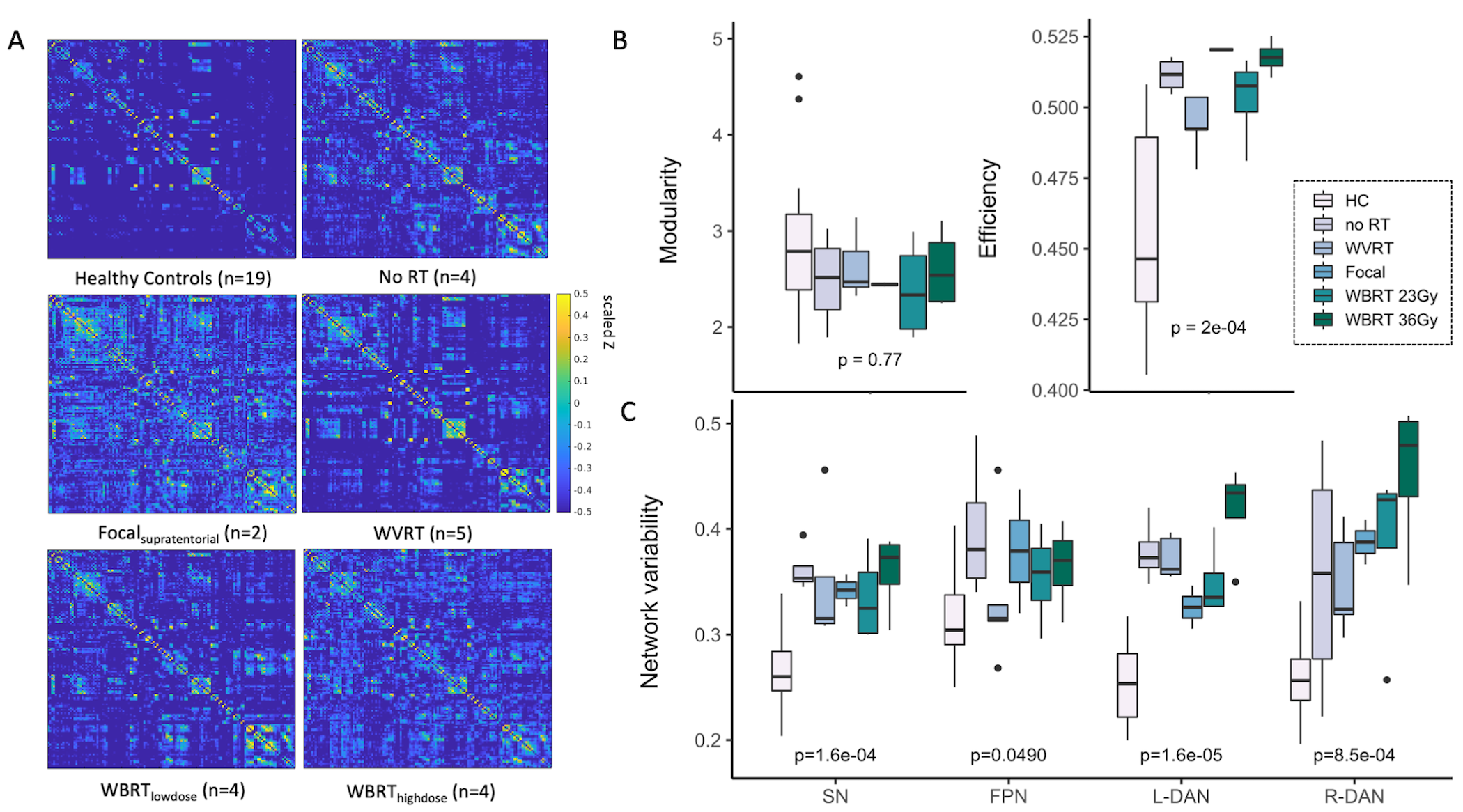

Supplement: Supplementary Figure 1 | — MR metrics can distinguish healthy controls and patient subgroups. Scaled and Fisher transformed functional connectivity (FC) matrices reveal notable group differences in global connectivity (A). MR metrics derived from FC matrices and ICA brain networks show significant group trends related to group exposure to radiation therapy (RT) and/or the degree of RT treatment aggressiveness (B, C). HC, healthy controls; no RT, non-irradiated patients; WVRT, whole-ventricular focal RT; Focal, focal RT to the supratentorial brain; WBRT, whole-brain RT. [file Image_1.tiff]
